# The role of autophagy-lysosomal pathway in motor neuron diseases

**DOI:** 10.1042/BST20220778

**Published:** 2022-09-16

**Authors:** Barbara Tedesco, Veronica Ferrari, Marta Cozzi, Marta Chierichetti, Elena Casarotto, Paola Pramaggiore, Francesco Mina, Margherita Piccolella, Riccardo Cristofani, Valeria Crippa, Paola Rusmini, Mariarita Galbiati, Angelo Poletti

**Affiliations:** 1 Dipartimento di Scienze Farmacologiche e Biomolecolari (DiSFeB), Università degli Studi di Milano, Milano, Italy; 2 Unit of Medical Genetics and Neurogenetics, Fondazione IRCCS Istituto Neurologico Carlo Besta, Milan, Italy

**Keywords:** autophagy, chaperone-assisted selective autophagy, HSPB8, motorneuron diseases, neurodegeneration, trehalose

## Abstract

Motor neuron diseases (MNDs) include a broad group of diseases in which neurodegeneration mainly affects upper and/or lower motor neurons (MNs). Although the involvement of specific MNs, symptoms, age of onset, and progression differ in MNDs, the main pathogenic mechanism common to most MNDs is represented by proteostasis alteration and proteotoxicity. This pathomechanism may be directly related to mutations in genes encoding proteins involved in the protein quality control system, particularly the autophagy-lysosomal pathway (ALP). Alternatively, proteostasis alteration can be caused by aberrant proteins that tend to misfold and to aggregate, two related processes that, over time, cannot be properly handled by the ALP. Here, we summarize the main ALP features, focusing on different routes utilized to deliver substrates to the lysosome and how the various ALP pathways intersect with the intracellular trafficking of membranes and vesicles. Next, we provide an overview of the mutated genes that have been found associated with MNDs, how these gene products are involved in different steps of ALP and related processes. Finally, we discuss how autophagy can be considered a valid therapeutic target for MNDs treatment focusing on traditional autophagy modulators and on emerging approaches to overcome their limitations.

## Introduction

Motor neuron diseases (MNDs) are a large class of neurodegenerative diseases (NDs) primarily affecting neurons that control the skeletal muscle fibers. These neurons comprise upper motor neurons (MNs) localized in the motor cortex and lower MNs localized in the bulbar regions or in the anterior horns of the spinal cord. Other cell types surrounding (e.g. glial and microglial cells) or connecting to (e.g. sensory neurons, interneurons, muscle cells, etc.) MNs may contribute to disease onset and progression, and ultimately to paralysis and death of the patients. These neural systems are governed by a complex architecture, thus various MN types are differentially and selectively involved in these disorders, leading to multifaceted clinical manifestations that considerably vary among affected individuals.

To simplify, MNDs can be roughly classified into three major categories that reflect both the clinical signs and the type of MNs affected: (i) with lower MNs involvement (e.g. spinal muscular atrophy (SMA), spinobulbar muscular atrophy (SBMA or Kennedy's disease), progressive muscular atrophy (PMA), pseudobulbar palsy (in the bulbar region), monomelic amyotrophy (MMA), lethal congenital contracture syndrome (LCCS), etc.), (ii) with upper MNs involvement (e.g. primary lateral sclerosis (PLS) and hereditary spastic paraplegias (HSP), etc.), and (iii) with combined upper and lower MNs involvement (e.g. amyotrophic lateral sclerosis (ALS), etc.) [1–3]. Overall, motor manifestations depend on which MNs are affected: lower MN loss results in muscle weakness, and upper MN loss in spasticity; mixed clinical signs associate initially to upper and lower MN loss, but when the disease progresses weakness and flaccid paralysis prevail over spasticity. Noteworthily, this basic classification does not fully reflect the existence of mixed forms, as, for example, ALS with frontotemporal dementia (FTD) [4].

No cure is available for most of these MNDs. Only for specific forms of SMA some highly innovative genetic approaches have recently proved to be therapeutically efficient [5–7].

The molecular bases of these MNDs may considerably differ and, while specific inherited (familial) forms are characterized by mutations in genes encoding proteins involved in a variety of intracellular and extracellular biological functions, some MNDs occur in a sporadic manner, making it very difficult to determine the causes of MN loss. Nevertheless, sporadic forms often display a dysregulation of the same proteins that have been identified mutated in familial forms of MNDs (see the case of sporadic and familial ALS) [8]. Intriguingly, many of these proteins and their mutated counterparts act in the neuronal response to proteotoxic stresses, which includes the protein quality control (PQC) system and particularly the autophagy-lysosomal pathway (ALP). ALP is essential to degrade damaged organelles or toxic protein aggregates and inclusions capable to alter cellular homeostasis causing MN death. This review will thus focus on the role of ALP in MNDs.

## The autophagy-lysosomal pathway

**Figure 1.**
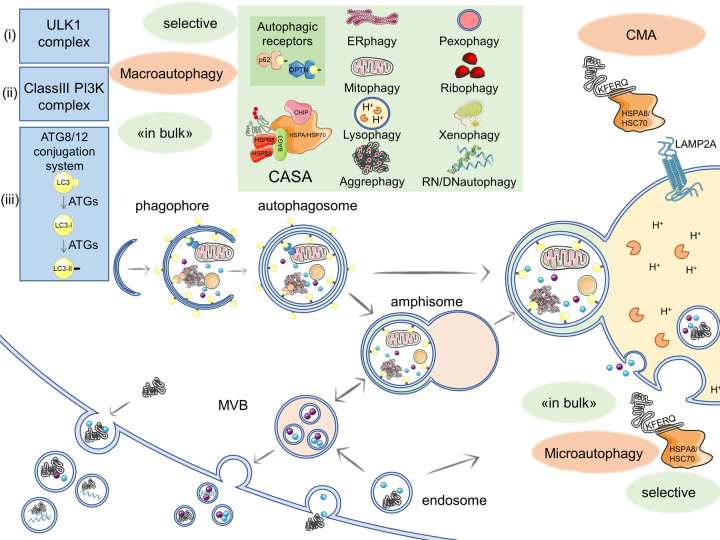
Overview of the autophagy pathway. Macroautophagy is finely regulated by ATGs and other proteins forming multimeric complexes responsible for (i) initiation (ULK1 complex, activated by various signals), (ii) phagophore formation (Class III PI3K complex), and (iii) elongation (ATG8/12 conjugation systems). In macroautophagy, cargoes are engulfed «in bulk» or selectively (green box), with the involvement of autophagic receptors (e.g. SQSTM1/p62, OPTN), into membranous structures (phagophore) which close forming a double membrane vesicle decorated with LC3-lipidated proteins (autophagosome), which fuses with the lysosome. In microautophagy, a direct invagination of the lysosomal membrane («in bulk») sequesters the substrates to be degraded or, alternatively, substrates carrying a KFERQ-like motif are recognized and directed to endosomes by the HSPA8/HSC70; in CMA, substrates carrying a KFERQ-like motif are bound by HSPA8/HSC70 and internalized into the lysosome in a LAMP2A-dependent manner. Autophagy pathway cross-talks with the endosomal system: substrates can be internalized into endosomes. Endosomes can be directly targeted to lysosomes or maturate in multivesicular bodies (MVBs) that fuse with autophagosomes forming amphisomes, which are addressed to lysosomal degradation. Substrates can be also cleared from cells through exocytosis by MVBs fusion with cell membrane or other vesicles budding. This figure was created using Servier Medical Art templates, licensed under a Creative Commons Attribution 3.0 Unported License; https://smart.servier.com [23].

The term ‘autophagy' refers to various cellular processes involved in proteins or organelles clearance via the lysosomal system. Autophagy eliminates unused, excessive, and/or defective macromolecules (either endogenous or exogenous) and recycles their components for other biosynthetic pathways (Figure 1). Autophagy takes place in the cell cytoplasm, and it is an essential arm of the PQC and RNA quality control system, which works together with the proteasome in a finely tuned equilibrium. The fate of a given substrate is regulated by a plethora of chaperone proteins, that define how substrates will be cleared from cells. Autophagy dysregulations cause several acute and chronic human diseases and promote the aging process; these findings have stimulated the search for autophagy modulators as potential therapeutic tools to efficiently counteract them [9–11].

Autophagy is a complex system, in which the delivery of cargoes to lysosomes for degradation follows different routes named (i) macroautophagy (usually referred to as autophagy), (ii) microautophagy, and (iii) chaperone-mediated autophagy (CMA).

Macroautophagy implies that cargoes are sequestered by ‘omegasomes' released by the endoplasmic reticulum (ER) or other membranous compartments (e.g. late endosomes, Golgi apparatus, or plasma membrane). Omegasomes release double-membrane structures to enclose substrates generating the autophagosomes. This process is tightly regulated and controlled by the autophagy-related proteins (named ATGs) which orchestrate autophagosome assembly, trafficking, and fusion to lysosomes [10,12]. The type of cargo determines whether macroautophagy occurs ‘in bulk' (i.e. cytoplasm portions engulfed into autophagosomes), or ‘selectively', thanks to specific autophagy receptors (e.g. sequestosome 1 (SQSTM1/p62), optineurin (OPTN), etc.) that bridge cargoes to the autophagosome membranes. This is enabled by the presence of an LC3-interacting region (LIR) in most of the autophagy receptors. The LIR allows them to interact with members of the LC3/GABA Type A Receptor-Associated Protein (GABARAP) family (e.g. the microtubule-associated protein light chain 3 (MAP-LC3 or LC3)) anchored in their lipidated form (LC3-II) on the autophagosomes surface [13]. Through this mechanism, damaged organelles or other substrates are selectively removed from the cytoplasm [14]. For instance, terms such as ERphagy, mitophagy, lysophagy, ribophagy, pexophagy, RN/DNautophagy, xenophagy, aggrephagy describe the selective removal of damaged structures of ER, mitochondria, lysosomes, ribosomes, peroxisomes, nucleic acids, pathogens, and protein aggregates, respectively (Figure 1). Once substrates have been engulfed into autophagosomes, macroautophagy ends with the fusion of autophagosomes with lysosomes for cargo degradation.

**Figure 2.**
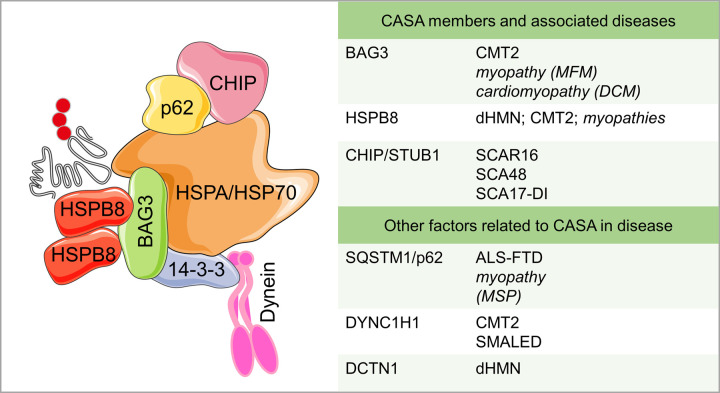
CASA involvement in MNDs and other neuromuscular conditions. The list reports factors of the CASA pathway of which mutations have been found in diseases affecting the neuromuscular system. In particular, BAG3 and HSPB8 mutations are described in neuropathies (CMT2 and dHMN) and myopathies. BAG3 mutations are also causative of dilated cardiomyopathy (DCM). CHIP/STUB1 mutations are associated with different forms of SCA. Other factors that participate in CASA pathway are SQSTM1/p62, mutated in ALS-FTD and MSP, the dynein machinery components DYNC1H1 and DCTN1, mutated in CMT2 and SMALED or dHMN, respectively. CMT2 = Charcot–Marie–Tooth type 2; dHMN = distal hereditary motor neuropathy; DCM = dilated cardiomyopathy; SCA = spinocerebellar ataxia; (SCA)R = recessive; (SCA)-DI = Digenic; MSP = multisystem proteinopathy; SMALED = spinal muscular atrophy with lower extremity predominance. This figure was created using Servier Medical Art templates, licensed under a Creative Common Attribution 3.0 Unported License; https://smart.servier.com [23].

In diseases characterized by the presence of misfolded proteins prone to aggregate (including MNDs), aggrephagy may utilize a specific form of cargo delivery to the microtubule organization center (MTOC), where aggresomes are assembled [15–19]. This route, known as chaperone-assisted selective autophagy (CASA) [20,21], is based on misfolded protein recognition by a heteromeric complex of the small heat shock protein B8 (HSPB8) and its obligatory partner Bcl-2-associated athanogene 3 (BAG3) [22,23]. Once formed, HSPB8/BAG3 associate to HSPAs/HSP70s bound to the carboxyl terminus of HSC70-interacting protein (CHIP or STUB1), a U-box-containing E3 ubiquitin-protein ligase that tags misfolded proteins with a polyubiquitin chain for autophagy receptor recognition (SQSTM1/p62 for CASA). This protein complex, named CASA complex, is then transported along microtubules to the MTOC by the dynein machinery bound to BAG3 [22,23]. Next, the autophagy receptors allow the LC3-II-mediated insertion into autophagosomes. The relevance of the CASA in cell homeostasis is underlined by the fact that, when mutated, most of its members cause neuronal or muscular disorders (see Figure 2 for details).

Regarding mammalian microautophagy, cargoes are specifically engulfed into lysosomes via the intervention of late endosomes. Protein recognition can be achieved either ‘in bulk' or (like CMA, see below) selectively thanks to a KFERQ-like motif in the target protein which is recognized by the constitutive form of HSPAs/HSP70s, the heat shock cognate 70 (HSPA8/HSC70) [24]. In microautophagy, HSPAs/HSP70s activities are mediated by an invagination of the late endosome membrane with the involvement of the endosomal sorting complex required for transport (ESCRT) machinery. Entrapped proteins and organelles can be then directly processed in late endosomes or after their fusion with lysosomes [9,25–27]. HSPA8/HSC70 plays a pivotal role also in CMA. In CMA, the HSPA8/HSC70-bound substrate is not subjected to endosomal engulfment. Instead, the HSPA8/HSC70 recognizes and unfolds the cargo that is directly routed to lysosomes where it is released to the CMA receptor LAMP2A, thus escaping the late endosomal engulfment [28]. In CMA, only the proteins possessing the KFERQ-like motif can be cleared [29], even if this motif is relatively frequent in mammalian proteins and there is the intriguing possibility to activate it via post-translational modifications (phosphorylation, acetylation) acting as an off/on switch to regulate protein degradation [24]. When the cargo is released from HSPA8/HSC70 it is associated to LAMP2A, which oligomerizes into the lysosomal membrane. Oligomerization promotes the translocation of the cargo to the lumen, where another HSPA8/HSC70 extracts the cargo allowing its degradation (Figure 1).

## Mutant genes encoding autophagy-related proteins are linked to MNDs

As mentioned above, in the past two decades, it emerged that many mutant genes causative of specific MND forms encode proteins differentially involved in the regulatory steps of autophagy.

**Figure 3.**
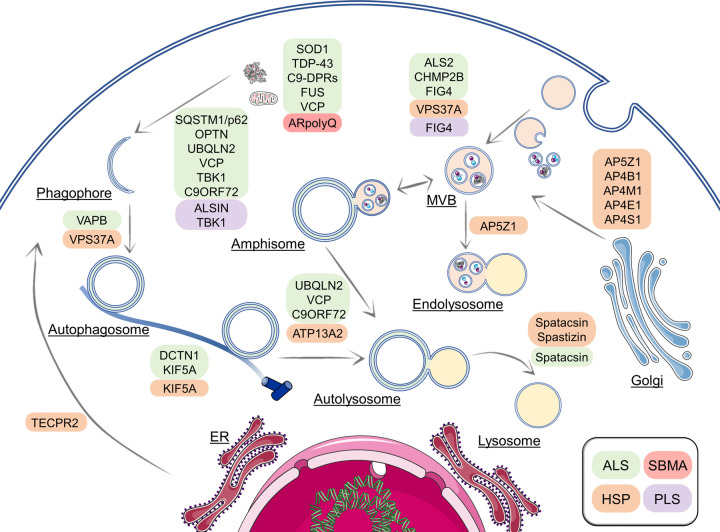
Schematic representation of the main genes involved in autophagy and endolysosome pathway associated to MNDs. In MNDs, various mutations have been identified in genes encoding for proteins involved in different steps of autophagy and endolysosome pathways which result impaired and dysfunctional. In parallel, mutant MNDs-related genes encode for proteins prone to misfold and aggregate which are substrates of autophagy. ALS = amyotrophic lateral sclerosis; HSP = hereditary spastic paraplegia; PLS = primary lateral sclerosis; SBMA = spinobulbar muscular atrophy. This figure was created using Servier Medical Art templates, licensed under a Creative Common Attribution 3.0 Unported License; https://smart.servier.com.

Indeed, by merely looking at the many different HSP forms, several mutated genes, called Spastic ParapleGia (*SPG*) genes, encode proteins that are key regulators of the endolysosomal system and autophagy. An autosomal dominant form of HSP is caused by a mutation of the *SPG42* gene encoding a protein involved in ER membrane transport, bone morphogenetic protein (BMP) signaling and autophagy [30–32]; other autosomal recessive forms are caused by mutant genes encoding proteins involved in autophagy as well as in lysosome shaping (e.g. *SPG11*, encoding spatacsin), in endosomal trafficking (e.g. *SPG15*, encoding spastizin), in vesicle formation and trafficking (e.g. *SPG47*, *SPG50*, *SPG51*, *SPG52*, *SPG53*, encoding AP4B1, AP4M1, AP4E1, AP4S1, VPS37A, respectively), in Golgi apparatus-multivesicular bodies (MVB) dynamics (e.g. *SPG48*, encoding AP5Z1), and lysosomal targeting to autophagosomes (e.g. *SPG49*, encoding TECPR2), or in membrane trafficking and mitochondrial function (e.g. *SPG78*, encoding ATP13A2) [30–34] (Figure 3 and Table 1). Mutations of these genes result through different mechanisms in MNs death and involve other neuronal populations, giving rise to the complex and mixed clinical manifestations which coexist with motor dysfunctions.

**Table 1.** Genes cited in this review involved in ALP

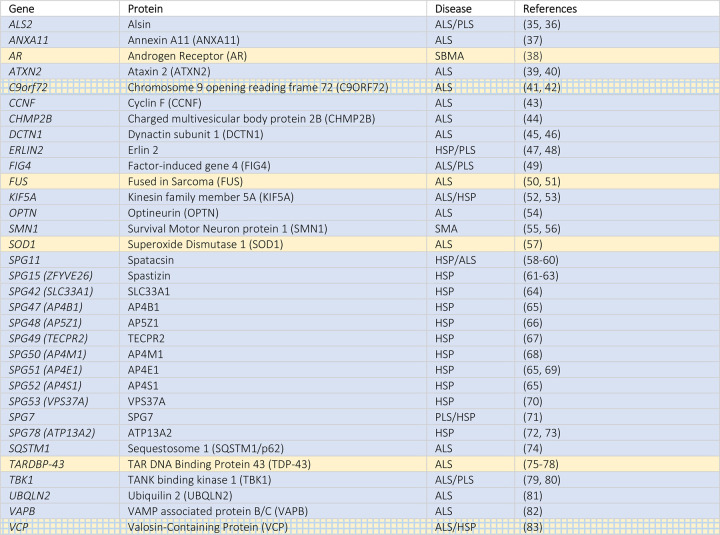

In blue, genes encoding proteins whose functions have been related to ALP. In yellow, genes that, when mutated, encodes protein products prone to misfold and aggregate, impairing proteostasis. Genes with both features are in yellow with blue lines.

**Figure 4.**
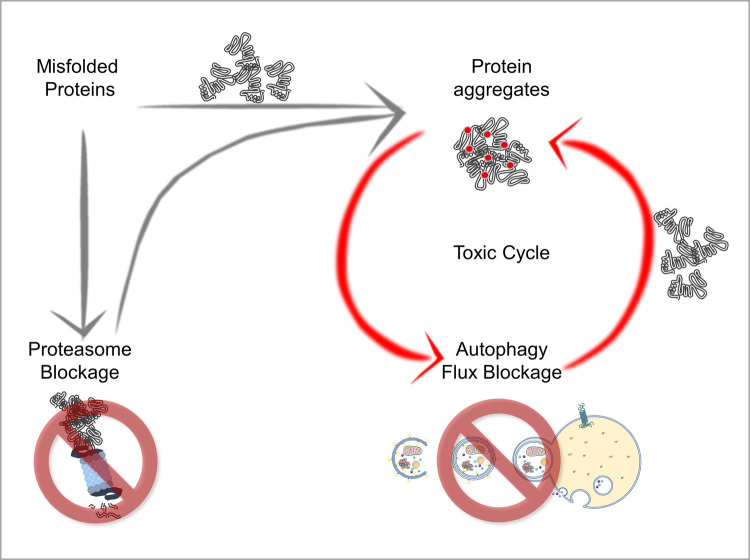
Toxic cycle nourishing proteostasis imbalance. An increased load in misfolded substrates may result in protein aggregates formation, which, in turn, hampers the autophagic flux. Impaired autophagy is not able to clear cells from misfolded substrates, which accumulate increasing the burden of misfolded and aggregating substrates. Proteasome saturation feeds this toxic cycle, worsening proteostasis imbalance.

Even more complex is the autophagy involvement in ALS [4,11,84]. As for HSP, several familial ALS forms are caused by mutations in genes encoding proteins regulating the endolysosomal and autophagic pathways (as extensively reviewed in [85]). Examples are the *C9ORF72* (encoding the C9orf72-SMCR8 complex subunit, guanine nucleotide exchange factor C9orf72, recently involved in autophagy), the *DCTN1* and *KIF5A* (encoding, respectively, dynactin subunit 1 (a component of dynein motor complex) and kinesin family member 5A, involved in the autophago-lysosome fusion process and, for dynein/dynactin, in the routing of the CASA complex to the MTOC), *VCP* (encoding the valosin containing protein, a hexameric complex involved in misfolded protein extraction for their degradation via different pathways, including autophagy), *SQSTM1/p62* and *OPTN* (encoding the Sequestosome 1 and the optineurin, two autophagy receptors), *TBK1* (encoding the TANK binding kinase 1, involved in the innate immune response, inflammation, cell proliferation responses, and autophagy), *FIG4* (encoding the factor-induced gene 4, a magnesium-dependent phosphatase converting phosphatidylinositol-3,5-bisphosphate (PI(3,5)P2) into phosphatidylinositol-3-phosphate (PI(3)P) on endosomal membranes, a process relevant for endosomal transport and autophagy), and others.

Moreover, several mutant genes encode proteins not directly involved in autophagy, but related to mechanisms that facilitate the process (e.g. genes encoding proteins involved in vesicle trafficking (and apoptosis, exocytosis, and cytokinesis), like *ANXA11*, encoding annexin A11; in endocytosis (and RNA metabolism), like *ATXN2*, encoding ataxin 2; in sorting of endosomal cargo proteins, like *CHMP2B*, encoding the charged MVB protein 2B, a component of the ESCRT complex III; in vesicle trafficking from the ER-membrane, like *VAPB*, encoding VAMP associated protein B/C) (Figure 3). Also, the molecular re-routing system from autophagy to proteasome and *vice versa* may indirectly cause autophagy alterations. In fact, this system maintains the fine-tuned equilibrium between these two degradative pathways. The best example is the routing system based on the activity of HSPAs/HSP70s-CHIP, which is differentially regulated by their selective association to HSPB8/BAG3 (CASA for autophagic degradation) or to BAG1 (for proteasome degradation) [22]. Autophagy could be required as an alternative mechanism, in case of mutations occurring in *UBQLN2* or *CCNF* (encoding, respectively, Ubiquilin 2 and cyclin F, the first physically associated with both proteasome and ubiquitin ligases and involved in protein degradation via proteasome, the second catalyzing ubiquitin transfer to substrates for proteasomal degradation), even if it is still unclear whether the autophagic response may compensate the altered proteasome pathway.

The impact of some of the mutations/variants reported above on ALS onset and progression is debated. A recent database (ALSoD (https://alsod.ac.uk/)) has tried to correlate how the variants are linked to ALS, even when evidence is weak.

It should be underlined that mutations in some of the genes associated to HSP and ALS may also be responsible for the appearance of PLS (e.g. *SPG7*, *TBK1*, *ALS2*, *ERLIN2*, *FIG4*) [86]. Similarly, a FTD phenotype may be present at different degrees in ALS cases (even as pure FTD with some ALS-related mutant genes). Therefore, it is difficult to draw clear correlations between genotype and phenotype in these diseases.

Even in the case of SMA, the causative survival MN (SMN1) protein (a protein implicated in spliceosome assembly and ribonucleoproteins biogenesis, mRNA trafficking, local translation, and cytoskeletal dynamics) has been found involved in endocytosis and autophagy (see [87] for review), although the mechanism by which SMN controls or is regulated by autophagy is still poorly understood [88–90].

## Mutant genes encoding proteins prone to misfold and aggregate cause MNDs

While some mutated genes causative of MNDs directly modulate ALP, many others indirectly impact on autophagy as a consequence of the aberrant biochemical properties acquired by the mutant protein. This occurs when a mutation modifies the native protein (i) altering its capability to properly fold, (ii) enhancing its hydrophobicity, (iii) affecting highly disordered domains, or (iv) altering its interaction with partners, etc. These mechanisms are not mutually exclusive and often increase the propensity of the mutant proteins to clamp together, forming aggregate structures of diverse nature: liquid–liquid droplets or fluid condensates, solid-like aggregates or densely packed insoluble protein inclusions, etc., based on their mechanism of formation and/or their stage of maturation. As a function of their biochemical state, aggregates can be dissolved or further processed to be cleared from cells via autophagy. However, aggregated proteins may impair the autophagic flux, causing severe consequences to the PQC system, with the degradative capability of affected neurons (or surrounding cells affected in MNDs) being overwhelmed. Indeed, this is the case of SBMA, caused by the transcription factor androgen receptor (AR) carrying an elongated polyglutamine tract (ARpolyQ) in its N-terminus. Soon after its translation, the polyQ is masked by specific chaperones (HSPAs/HSP70s, HSP90, etc.), that are released upon AR-binding with its natural androgenic ligands. These molecules activate the AR, allowing conformational changes that unmask the polyQ, causing ARpolyQ aggregation and impairment of the autophagic process [91–98]. Despite a deep investigation, it is still not totally clear whether aggregates cause autophagic flux blockage, or if a defective autophagy results in poor protein clearance and thus excessive protein aggregation. Likely, the two processes equally contribute to generate a toxic cycle in which defective autophagy allows protein accumulation, that in turn, perturbs the autophagic pathway, potentiating the deleterious effects of ARpolyQ in MNs (Figure 4). This toxic cycle could also involve the proteasome system, which has a low degradative capacity and only processes monomeric unfolded proteins. Thus, when the proteasome is saturated by an excessive amount of substrates to be degraded, these accumulate worsening the autophagic flux blockage previously mentioned [99–107]. Similar processes take place also in the case of various mutant proteins causative of familial ALS or HSP. In fact, like the ARpolyQ in SBMA, most of these mutated proteins tend to misfold and aggregate (e.g.: Superoxide Dismutase 1 (SOD1), TAR DNA-Binding Protein 43 (TDP-43, and its fragment of 35 and 25 kDa), fused in sarcoma (FUS), VCP, the dipeptide repeats (DPRs) products of the C9ORF72 mRNA, etc.) [100,108–129], possibly impairing the degradative systems including autophagy. Notably, the exogenous expression or a pharmacological transcriptional induction of HSPB8 [22,116,117,130], both robustly enhance mutant misfolded and aggregated protein clearance. Since HSPB8 is not an autophagy inducer, its enhancement facilitates the delivery of misfolded proteins along microtubules for their autophagic clearance [104,131–134], improving or restoring a normal autophagic flux.

## Autophagy modulation as therapeutic tool in MNDs

Based on what reported above, autophagy activation may represent a valuable therapeutic approach for MNDs, but this approach has to be carefully evaluated considering the specific alteration present in each clinical form studied.

For example, in case of insufficient autophagy (e.g. lack of crucial factors linked to haploinsufficiency in MNDs associated with loss-of-function mutations; recessive forms involving proteins which can be replaced by the activity of other factors, etc.), it might be useful to stimulate the remaining functional wild type allele, or ‘redundant' factors to restore autophagy. Unfortunately, autophagy and its regulation may considerably differ in the various cell types affected in MNDs (neurons, MNs, glial cells, microglia, muscle cells) making it difficult to identify the proper therapeutic agent for each MND form [11].

In general, drugs stimulating autophagy are relatively poorly specific. This is the case of the mammalian target of rapamycin (mTOR) inhibitor rapamycin, tested in MND animal models [135] and under investigation in ND patients [136,137], that acts not only on autophagy, but on a plethora of other intracellular pathways. Lithium has also been tested in some forms of MNDs, even if its activity as an autophagy inducer is rather controversial [138–141].

Other autophagy inducers, like trehalose and the more stable derivatives melibiose and lactulose, appear to be more promising, since their molecular mechanism of action is more selective and impacts directly on the autophagy master regulator transcription factor EB (TFEB) [104,142–146]. Trehalose has already been successfully tested in several animal models of NDs [147–159]. No double-blind clinical trial has been started yet, even if a single-arm, open-label pilot study has been recently reported on Niemann–Pick disease types A and B patients to assess its potential efficacy after intravenous delivery for 3 months. Despite the size of the study was small, a positive (not statistically relevant) trend was reported [160], suggesting that larger studies are needed to verify trehalose efficacy.

In the case of some MND forms in which autophagy is already enhanced in response to the overload of aberrant aggregation-prone proteins, generic autophagy activators may not be effective treatments. Instead, the pharmacological targeting of factors specifically acting on misfolded protein recognition and delivery to autophagy will represent a valid therapeutic strategy. The drug colchicine has these properties since it has been identified as a potent activator of the human HSPB8 promoter in MNs. Its capability to enhance HSPB8 expression was correlated with an increased CASA-mediated removal of ALS-associated proteins (TDP-43 and its disease associated fragments) in ALS cell and fly models [161]. A double-blind clinical trial with colchicine is presently ongoing on a group of ALS patients [133].

## Conclusions

In conclusion, autophagy is an essential cellular process that maintains proteostasis in neurons and other cell types typically affected in MNDs. Its role in MNDs is extremely complex as both its dysregulation and its insufficient or excessive activity play multiple roles in the appearance and/or progression of different forms of MNDs and other NDs. Moreover, it is still unclear why this relatively conserved mechanism among the various cell types of the human body associates to so many different pathological conditions. Since these diseases are characterized by the selective vulnerability of different neuronal populations, other modifier factors might differentially contribute to exacerbate or attenuate, respectively, the deleterious or protective activities of autophagy in each neuronal type. Therefore, the identification of a proper target to be tackled by drugs capable of modulating the autophagic pathways is still challenging. However, it is emerging that approaches aimed to facilitate an already activated autophagy (e.g. those acting on the selective delivery of misfolded proteins to the autophago-lysosomes for their clearance) will provide more tailored treatments to counteract the neurotoxicity of aggregation-prone proteins in a wide variety of MNDs linked to defective response to proteotoxic stimuli.

## Perspectives

- MNDs comprise several untreatable neurodegenerative diseases characterized by the loss of neurons controlling voluntary movements. Several MND forms are associated to alteration of the main degradative systems, particularly the ALP.
- Several genes found causative of MNDs are involved in the regulation of the various forms of autophagy. Other genes code proteins that perturb proteostasis in neurons indirectly altering autophagy.
- Different compounds have been developed to stimulate and/or facilitate the ALP, some have already been successfully tested in animal models of MNDs and may be utilized soon for advanced clinical trials in MND patients.

## Abbreviations

ALP: autophagy-lysosomal pathway
ALS: amyotrophic lateral sclerosis
ANXA11: annexin A11
AR: androgen receptor
ATGs: autophagy-related proteins
ATXN2: ataxin 2
BAG3: Bcl-2-associated athanogene 3
BMP: bone morphogenetic protein
CASA: chaperone-assisted selective autophagy
CCNF: cyclin F
CHIP/STUB1: carboxyl terminus of Hsc70-interacting protein
CHMP2B: charged multivesicular body protein 2B
CMA: chaperone-mediated autophagy
DCTN1: dynactin subunit 1
DPRs: dipeptide repeats
ER: endoplasmic reticulum
ESCRT: endosomal sorting complex required for transport
FIG4: factor-induced gene 4
FTD: frontotemporal dementia
FUS: fused in sarcoma
GABARAP: GABA Type A Receptor-Associated Protein
HSP: hereditary spastic paraplegia
HSPA8/HSC70: heat shock cognate 70
HSPAs/HSP70s: heat shock protein 70
HSPB8: heat shock protein B8
KIF5A: kinesin family member 5A
LAMP2A: lysosome-associated membrane protein type 2A
LCCS: lethal congenital contracture syndrome
LIR: LC3-interacting region
MAP-LC3: microtubule-associated protein light chain 3
MMA: monomelic amyotrophy
MNDs: motor neuron diseases
MNs: motor neurons
MTOC: microtubule organization center
mTOR: mammalian target of rapamycin
MVBs: multivesicular bodies
NDs: neurodegenerative diseases
OPTN: optineurin
PI(3)P: phosphatidylinositol-3-phosphate
PI(3,5)P2: phosphatidylinositol-3,5-bisphosphate
PLS: primary lateral sclerosis
PMA: progressive muscular atrophy
polyQ: polyglutamine
PQC: protein quality control
SBMA: spinobulbar muscular atrophy
SMA: spinal muscular atrophy
SMN: survival MN
SOD1: superoxide dismutase 1
SPG: spastic paraplegia [gene]
SQSTM1/p62: sequestosome 1
TBK1: TANK binding kinase 1
TDP-43: TAR DNA-binding protein 43
TFEB: transcription factor EB
UBQLN2: ubiquilin 2
VAPB: VAMP associated protein B/C
VCP: valosin containing protein
